# Challenges and Potential Opportunities of Mobile Phone Call Detail Records in Health Research: Review

**DOI:** 10.2196/mhealth.9974

**Published:** 2018-07-19

**Authors:** Kerina Helen Jones, Helen Daniels, Sharon Heys, David Vincent Ford

**Affiliations:** ^1^ Data Science Building School of Medicine Swansea University Swansea United Kingdom

**Keywords:** call detail records, mobile phone data, health research

## Abstract

**Background:**

Call detail records (CDRs) are collected by mobile network operators in the course of providing their service. CDRs are increasingly being used in research along with other forms of big data and represent an emerging data type with potential for public good. Many jurisdictions have infrastructures for health data research that could benefit from the integration of CDRs with health data.

**Objective:**

The objective of this study was to review how CDRs have been used in health research and to identify challenges and potential opportunities for their wider use in conjunction with health data.

**Methods:**

A literature review was conducted using structured search terms making use of major search engines. Initially, 4066 items were identified. Following screening, 46 full text articles were included in the qualitative synthesis. Information extracted included research topic area, population of study, datasets used, information governance and ethical considerations, study findings, and data limitations.

**Results:**

The majority of published studies were focused on low-income and middle-income countries. Making use of the location element in CDRs, studies often modeled the transmission of infectious diseases or estimated population movement following natural disasters with a view to implementing interventions. CDRs were used in anonymized or aggregated form, and the process of gaining regulatory approvals varied with data provider and by jurisdiction. None included public views on the use of CDRs in health research.

**Conclusions:**

Despite various challenges and limitations, anonymized mobile phone CDRs have been used successfully in health research. The use of aggregated data is a safeguard but also a further limitation. Greater opportunities could be gained if validated anonymized CDRs were integrated with routine health records at an individual level, provided that permissions and safeguards could be put in place. Further work is needed, including gaining public views, to develop an ethically founded framework for the use of CDRs in health research.

## Introduction

### Background

There are already over 5 billion unique mobile device subscribers globally, and the number of mobile connections exceeds the world population at over 8 billion [1]. Mobile phone penetration is constantly rising and is predicted to exceed 5 billion users by 2019 [2]. As mobile phones are now an integral part of modern life, their potential to be used as a means of improving health care is increasingly promising. Call Detail Records (CDRs) are collected by mobile network operators (MNOs) in the course of providing their service. Each time a mobile phone user connects to a mobile network, either by voice call or text message, a record is generated that includes the starting time of the call (or message), its duration, the caller and receiver phone numbers, and their locations [3]. Locations are estimated from the positions of activated cell towers and can be made more precise via tower triangulation and Wi-Fi connections [3]. Unlike interaction with mobile phone apps, CDRs result from passive data collection requiring no additional effort by the end user. MNOs receive billions of CDRs globally; they are necessary for billing, monitoring data usage, and for understanding and targeting customers according to their mobile phone use [3].

Due to the lack of landline infrastructures, mobile phones are the preferred method of communication in low- to middle-income countries (LMICs) [4], and they are playing a crucial part in these countries’ socioeconomic developments [5]. Using mathematical modeling techniques, researchers are able to use CDRs to estimate the location of different populations and how this changes over time. Information on migratory patterns within and between countries can offer valuable information to policy makers in areas such as agriculture, transportation, poverty, conflict prevention, and disaster response and humanitarian aid [6]. In the last 5 years, CDR data have been used to improve health and health care, for example, via the generation of epidemiological models that can infer the spatial spread of infectious diseases from human mobility patterns [7].

Unlike for some of these LMIC settings, many developed countries have created data-intensive health research infrastructures, integrating multiple sources of anonymized routine health and administrative data for secondary uses. Main models vary between centralized repositories [8,9] and federated systems with distributed data nodes [10,11]. Typically, in a repository model, data are centralized for integration, whereas in a federated model data remain distributed among their original sources, with various permutations on these models and on how a user accesses data. Data provision to researchers can entail, subject to information governance regimes, external release of linked data to researchers [12], access via a data-safe haven and release of results [8], or by using privacy-preserving distributed data mining that computes distributed data without revealing sensitive information [13,14]. Address-based grid reference location data used with routine data present additional opportunities by enabling health geography studies but also present particular disclosure risks that must be mitigated so the data can be used safely [15]. CDRs represent an alternative type of spatial data that could add to, or augment, routine data collections to enable new opportunities for health research.

### Objective

The objective of this study was two-fold: (1) to review the ways in which CDRs (particularly the location elements) have been used in health research to identify the challenges encountered and benefits gained, and (2) to use this information to explore the issues that would need to be addressed to enable wider use of CDRs for health research, including their integration with routine health and administrative data.

## Methods

A literature review was conducted using structured search terms making use of major search engines. Predetermined eligibility criteria were set and adhered to in order to avoid the introduction of bias and to preclude the selection of studies on the basis of whether they favored a particular conclusion. To be included in the review, studies must have been published in the English language and in either peer‑reviewed journals or conference proceedings. Studies must have used CDR data to answer a research question. Therefore, methodological papers, for example, outlining different mathematical approaches for analyzing mobile phone datasets, were excluded. Studies using data derived from mobile phone apps were also excluded. Research on any study population and health-related condition was included.

A search strategy was devised based on these inclusion criteria aided by identifying keywords from seminal works in this field [3]. The search terms were as follows: (mobile phone location data) OR (mobile phone call data) OR (mobile phone data) OR (cell phone data) OR (cell phone call data) OR (cell phone location data) OR (call detail records). These keywords were chosen in order to conduct a sensitive, rather than a specific search to ensure a higher probability of including all relevant articles. The strategy was customized according to the stipulations of each database for building search strings. Searches covered studies published up until January 2017 with no restrictions on the earliest date of publication. The following databases were searched from February 7-25, 2017: the Cochrane Central Register of Controlled Trials (CENTRAL) [16], Google Scholar [17], PubMed [18], Scopus [19], Web of Science [20], and WorldPop [21]. It was not intended as an exhaustive review, but we took a pragmatic approach to identify a comprehensive range of health-related studies to identify benefits and challenges with reasonable confidence.

All search results were imported into an online reference generator [22] and duplicate references were removed. The title and abstracts of these results were screened against the inclusion criteria as outlined above to identify potentially eligible studies for which the full texts were reviewed. Two reviewers independently performed the search, and disagreements between the reviewers were resolved by consensus. The reference lists of the eligible articles were searched to identify additional studies.

The following data were extracted for each eligible study: author, year published, title of article, country of population studied, research topic area, how datasets were used, findings, information governance, and limitations of the data.

## Results

### Overview

**Figure 1:**
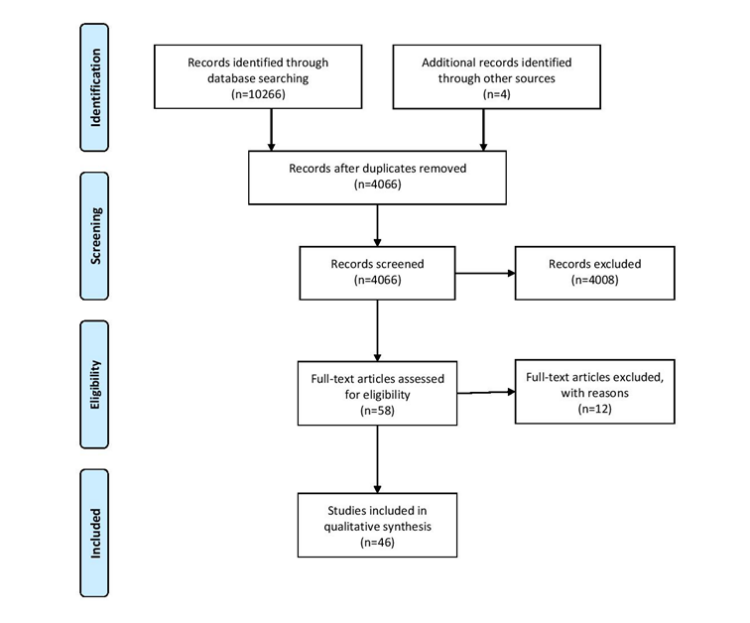
Preferred Reporting Items for Systematic Reviews and Meta-Analyses (PRISMA) diagram.

The search initially identified 4066 studies. Of these, 4008 were excluded on the basis of title and abstract alone as they did not meet the inclusion criteria. Most of the studies initially excluded tended to not be either research studies or health-related. Fifty-eight full-text articles were assessed for eligibility, and a further 12 were subsequently rejected for not meeting the inclusion criteria. Of these, two papers were excluded as they were not research studies, but rather described anonymization processes; four were excluded as they did not use CDR data; five were excluded as they were geographical studies but not on health-related research; and one study was excluded as it was a methodology paper. Figure 1 shows the study process flowchart according to Preferred Reporting Items for Systematic Reviews and Meta-Analyses (PRISMA) guidelines [23]. As a result of the screening process, 46 full-text articles were included in the qualitative synthesis. Multimedia Appendix 1 presents the included studies [24-69]. Studies using CDR data for health research are summarized. CDR data were used alone or in conjunction with additional datasets for a variety of health-related purposes. In each study, CDR data were used in mathematical modeling to predict or identify population movement or to construct and predict social networks. A narrative description, based on the information provided in the publications, is given here to provide further details and draw out notable issues.

### Research Topic Area and Findings

As is evident from Multimedia Appendix 1, the majority of studies (n=42) focused on LMICs, with the other four using data from Belgium (n=2), Austria, and Italy. By using mathematical modeling, CDR location data were used to predict the movement of a population. Often, CDR data were used to develop models to address pressing concerns on infectious disease transfer. These included (numbers of studies shown where >1) malaria (n=11), HIV (n=3), cholera (n=3), influenza (n=3) dengue fever, Ebola virus, schistosomiasis, Rubella, meningitis, and tuberculosis. Other studies made use of CDR data to model population movement after disasters, to design and target public health interventions by identifying the location of at risk populations, to model hospital catchments, to model effects of air quality, and to explore options to arresting infectious diseases at outbreak. Where additional datasets were used, these were overlaid and compared with existing CDR data. For example, virological data were used to understand how cases of influenza were spreading. These were then used to verify mathematical models generated using CDR location data by mapping how populations move [27]. Although this is a method of integrating data, none of the included studies actually linked CDRs on an individual level to other datasets.

### Datasets and Data Governance

Thirteen of the articles were on studies from the 2013 Orange Data for Development Challenge on Mobile Phone Data focused on Ivory Coast [70]. Orange is a large, international mobile network operator, who allowed international research laboratories access to anonymized CDRs generated by 5 million of their Ivory Coast customers dating between December 2011 and April 2012. Researchers were tasked to use the data in a way that could potentially contribute to the socioeconomic development of the country. CDRs were anonymized by Orange Ivory Coast and processed by Orange Labs in Paris. In addition, the geographical locations of the mobile phone masts were blurred to protect the commercial interests of Orange. These datasets were then released to researchers [70].

Four datasets of varying granularity were provided by Orange:

1. The number and duration of calls between a pair of antennae aggregated by each hour. These data were provided for the whole of the observation period.
2. High spatial resolution data (which published antenna identifiers) of individual movement trajectories. To reduce the possibility of identification, these data were supplied on a random sample of 50,000 individuals for a 2-week time period only.
3. Data for the entire observation period but with reduced spatial resolution to mitigate the risk of identification. Spatial resolution was reduced by using the subprefectures of the mobile phone antenna location rather than specific antenna identifiers.
4. Social network subgraphs using call data generated by 5000 randomly selected individuals. These were divided into 2-week time periods for the entire duration of observation [70].

A year later, Orange issued a second challenge, this time using data from Senegal [71]. Twelve of the studies included in this review used the data provided by this challenge. The data were pseudonymized locally in Dakar by Sonatel in the first instance. Orange Labs in Paris then undertook several layers of anonymization on the data. As with the Ivory Coast data, the true geolocations of mobile phone masts were masked. Again, to reduce the risk of identification, datasets of high granularity were restricted in time span, and coarser aggregated data covered longer periods of time [72]. An internal ethics workgroup reviewed the governance of each application to receive access to the data. An additional safeguard was also put in place for this particular challenge. An external ethical review panel was set up that consisted of 14 international members who provided the Orange team with advice on information governance, particularly to review risks in publishing findings. As well as privacy concerns, the external review panel considered political concerns and issues of civil unrest (eg, regarding the Ebola epidemic).

Of the studies not involved with either of the Orange Data for Development challenges, all studies apart from three [38,39,64] stated that the mobile phone CDRs they used in their research were anonymized. Few studies described the anonymization process as this was usually undertaken by the MNO beforehand. However, we have assumed, at the very least, that personal identifiers were removed from the data. MNO anonymization was not carried out in in one study [32] where researchers were provided with a raw dataset and undertook the anonymization process themselves. High- and low-volume users were excluded from analysis in this study to protect privacy. A second dataset from a different provider was used by the researchers in this same study. This dataset had been anonymized by the MNO: identifiable information was replaced by a hashed ID and encryption keys were exclusively managed by the MNO.

Wilson et al [67] stated that they followed the Groupe Spéciale Mobile Association (GSMA) privacy guidelines that advise that any analyses on mobile phone records should be done using de‑identified data and that individual level data should not leave MNO servers [73]. All analyses conducted by this team were performed by connecting remotely to a Linux server with only aggregated data transferred outside the operator.

Wesolowski et al [66] detailed how they complied with the laws and regulations of Pakistan and the MNO Telenor, by using only data aggregated to tehsil level (an administrative unit of Pakistan). This was processed on their behalf by Telenor employees. The following measures were implemented to preserve the privacy of Telenor Pakistan’s customers: (1) the CDR/mobility data were processed on a back-up and recovery server made available by Telenor Pakistan, with only Telenor employees having access to the detailed CDR/mobility data, (2) given the server arrangements, no detailed CDR/mobility data were taken out of Pakistan or left the premises of Telenor Pakistan, and (3) the processing of the detailed CDR/mobility data resulted in aggregations of the data on a tower-level granularity that was accessed only by Telenor employees.

Eight other studies mentioned explicitly that they used aggregated data in their analyses. There was no evidence to suggest that studies had a higher tendency to use aggregation if additional datasets had been used, but all additional datasets containing individual level data were de-identified. Three studies explicitly stated that they sought ethical approval from their own institution before beginning their research [28,36,57].

### Data Limitations

Several researchers commented on the limitations of the data, some of which are common to many data types and some specific to phone data. Gavric et al [41] reported that using aggregated data due to privacy concerns decreased the precision of their analyses. Differential mobile phone ownership due to financial means and socioeconomic status was noted as a potential source of bias whereby different sectors of a population may be over- or underrepresented [32,37,45,60]. For countries within Africa in particular, phone sharing is common, thus creating another potential source of bias since the network tracks the subscriber identification module (SIM), not the person. This would limit the value of studies focused on particular groups rather than general population movement [60].

## Discussion

### Principal Findings: Opportunities and Challenges

Emerging data types, such as mobile phone CDR data present valuable new opportunities for health research, particularly when used in combination with additional datasets. This study reports on a structured literature review on the use of CDR data for health research, showing that the immense volumes of CDR data collected and held globally by MNOs can be used for public health benefit. Of particular interest is the location element that can be used to track phone user movement at various levels of granularity. Most of the studies included in this review used CDR data to create mathematical models based on population movement to predict the spread of disease epidemics. Where additional datasets were used, often these verified the validity of these models.

Using CDRs in health research has a number of benefits. These data are routinely and passively collected via mobile phones without any effort needed by the end user. It could be argued that creating these big datasets in this way is far easier and more effective than recruiting and consenting participants into a research study individually. Also, CDRs can be generated from basic mobile phones and do not require the use of smartphone. Therefore, their use in research does not preclude those from low socioeconomic groups in the way that GPS data would, for instance. An individual’s home and work setting can be derived from CDR data, which is particularly valuable for countries where no integrated infrastructures exist for population census [74]. However, there are limitations that need to be taken into account in evaluating the use of CDRs in health research and in considering opportunities for their wider use. Data availability, formats, and levels of aggregation vary with MNO, and this will influence the type of analysis that can be done. For studies that require CDRs to be collected by multiple operators, researchers face the issue of having to join anonymized datasets: a difficult, although not insurmountable task [75]. There is an unknown level of discrepancy between phone owner and main user when phones are shared or bought for another person. This can call into question the validity of study findings focusing on particular demographics, since the extent of group representation is not known. Differential ownership of mobile phones among different sectors, particularly in LMICs where much of CDR research takes place, also calls into question the representativeness of the data and thus the findings that ensue [76]. However, it has also been observed that despite biases, there are few, if any, data sources that can provide such rich spatial and temporal movement data, particularly for much-needed research in LMICs [63,66].

A number of common patterns in data governance emerged from the review. Datasets were provided to researchers at different levels of spatial granularity and over variously restricted time periods to mitigate disclosure risks. These varied by study, or by programs of study. In some cases, data were subjected to several layers of anonymization and the true geolocations of mobile phone masts were masked. The use of anonymized (or strongly pseudonymized) data was the norm, with few studies outside this model and with many additionally using aggregation and the suppression of rare or extreme records. Reports of formal ethical review varied, but proposals were routinely submitted to an internal ethics workgroup, and in some cases to an independent external group for wider considerations such as political implications, societal benefits, and risk versus utility.

Concerns have been published on the ethics of using mobile phone data in research and the potential threats to privacy. Although MNOs have legal and organizational policies and researchers have jurisdictional health research governance to abide by, there is an absence of a clear, holistic, ethical, and regulatory framework to guide research using CDRs [3]. Most published research reports use anonymized data and many go further to protect against re‑identification via aggregation. Nevertheless, examples of how anonymization and aggregation do not guarantee privacy in location data are abundant in the literature [77-79]. Furthermore, breaches in group privacy do not rely on the re-identification of individuals. It is considered that individuals who belong to certain groups on the basis of their gender, sexual orientation, ethnicity, or political preferences could become visible in CDR data [80]. There is clearly a need for an ethically founded framework for the use of CDRs in research for public good.

Considering that CDR data are collected about members of the general public, such a framework should take into account public viewpoints on the use of CDRs for research. Public engagement is, of course, a common feature in health research and in the use of large-scale, anonymized person‑based data [81,82]. But it is acknowledged that similar work is both lacking and needful in the case of CDR data [3]. Although there have been surveys of public views on other aspects of mobile phone usage [83], there is no known literature on public perceptions of using CDRs for health research. Research using CDRs is still in its relative infancy, which means it is an ideal time to engage with the public so that their views can be taken into account in developing an ethically founded framework. This can be compared to similar work that has been done on genomic data sharing, another controversial area of health data science, where strong public views have necessitated the development of clear data sharing policies (eg, the Global Alliance for Genomics and Health [84]) [85-87].

### Wider Use and Future Work

From the review conducted in this study, plus seminal studies and reports [3,88], it is clear that CDR data can be valuable in health-related research. But as the majority of studies have focused on LMICs, the question arises of whether this can be translated into wider use in more developed countries, in particular where there are data-intensive infrastructures for health research that already have (or can gain) access to more traditional geolocation data, in the form of verified address-based grid references. Basically, it is a question of whether CDR data is valuable for health research per se, or only in particular settings. In support of the former position, some of the studies reviewed focused on Belgium, Austria, and Italy. Furthermore, Orange is not the only mobile network operator whose CDR datasets are being applied to health-related issues, as Telefonica have demonstrated via their Smart Steps initiative in various countries [89]. It may be that CDR data will prove to be a valuable resource for the public health sector, where, for example, the location element of the data could shed light on the way health promotion campaigns impact hospital attendances. Used in conjunction with additional datasets (eg, air quality monitoring databases, infection/virus outbreak data, emergency department attendance records), CDR location data may bring about new opportunities for health research. However, during the course of this study, a number of issues have been revealed that impact on opportunities for the wider use of CDRs for health research and that can be learned from in moving forward. These are considered here in relation to their integration with other relevant datasets for use in data‑intensive infrastructures, but they are also relevant to smaller‑scale health research endeavors.

An essential and primary issue is data availability since CDRs reside with the network operator. This alone could be a show-stopper for various reasons, including that GSMA guidance states that individual level CDR data should not leave MNO servers [73,90]. Assuming data could be accessible, there would need to be suitable mutually beneficial agreements between the MNO and research program owner, with the prerequisite of a shared vision for data use [88]. Following modeling exercises in conjunction with Vodafone, the UK Office of National Statistics is planning to use CDRs to monitor commuter travel and collect census data [91]. There will, of course, be a financial cost for the use of the data, the extent of which is not known. But to be viable, this will need to be lower than current costs and/or provide valuable new information. Cost is a second major operator‑related issue that may preclude the wider use of CDRs in health research unless suitable collaborative arrangements can be made.

Limitations inherent in the data (outlined earlier) need to be quantified and addressed for greater confidence in research findings. Encouragingly, there are reports that propose how bias in phone data can be addressed [74], but other issues remain. It would be useful to see a series of studies that have assessed the validity of CDRs in health research compared to other forms of geolocation data, for example, with Bluetooth data [92], photographic data [93], and flight data [94]. Such efforts would strengthen the evidence base on whether investment in the wider use of CDR data is warranted.

As with the use of any person-based data type, suitable physical, technical, and procedural controls need to be agreed between the data provider (MNO) and data user, as part of a proportionate data governance regime. This is true, though the stipulations may differ, whatever infrastructural and data access models are in place. The fact that anonymized and even aggregated data can pose identity disclosure risks is beyond doubt. Disclosure risks in the use of CDRs have not been studied to the same extent, and further work would be beneficial [15]. Ideally, this should be done in collaboration with an amenable network operator and be based on integrating CDRs with health records. However, this could be a challenge for reasons already described, but modeling a variety of data use scenarios using metadata (if available) could be a useful compromise and still yield meaningful information. This, along with the identified need for the input of public views on the use of CDR data for health research, would also inform the much needed consistent, holistic, ethically founded framework.

Public engagement forms an important part of an ethical framework for data use, beyond strict legal compliance requirements. Consultation with the public to gain their views on the use of CDRs for health research would gauge knowledge and expectations and, as with other emerging data types, would inform the socially acceptable use of the data. This is not too much to ask, since after all, CDRs originate with individuals and are based on their activities. It would be interesting to know more about the public’s actual awareness of data collected by mobile operators and the ways data are used in-house and by external agencies.

A last consideration is of the ultimate feasibility of integrating CDRs with health record data in terms of whether there is an appetite for it among the participating stakeholders. This is not a question that can be answered easily without seeing how the many challenges could be addressed so that the risk versus utility of such a development could be evaluated. A definite appetite and strong drivers to move initiatives forward will be needed for success.

### Strengths and Limitations

This study adds to the discussion on the suitability of CDRs for health research and raises issues to be addressed if the wider use of CDRs is to become a reality. It is the only known study to carry out a review of publications using CDRs in health research in order to learn from their practices and identify challenges. It also considers the use of CDRs in relation to data-intensive infrastructures and sets out problems to be solved to enable informed decisions on whether investment in seeking CDR integration is warranted. In accordance with other authors, this study recommends the development of an ethically founded framework for the use of CDRs in health research, but furthermore, it recommends that public views on the use of CDR data should be integral.

However, there are some limitations to consider. This study focused on articles using CDRs for health-related topics published in peer-reviewed journals and conference proceedings; it does not claim to be an exhaustive review. The use of health monitoring apps is outside the scope of the study, like Ginger.io [95] and the work of Sandy Pentland who, for example, used behavioral information from voice recordings and texting to detect signs of posttraumatic stress disorder in returning soldiers [96]. It also drew on reports published by MNOs and other organizations. However, operators will also have proprietary information not shared publicly, which may contain planned developments to address pertinent issues and take forward the use of CDRs for public benefit.

### Conclusion

All things considered, there are possibilities for the wider use of CDR data in health research but there are also major challenges to be addressed. Some important points have been discussed here, but this is not an exhaustive list of issues. Questions remain around the suitability of CDRs for wider use in health research and particularly as part of data-intensive infrastructures for population‑scale studies. A concerted effort will be needed to create solutions to determine if mobile phone CDRs are a worthwhile data type to pursue and invest in to augment currently used geolocation data.

## Appendix

Multimedia Appendix 1 Summary of included studies.

## Abbreviations

CDR: call detail record
GSMA: Groupe Spéciale Mobile Association
LMIC: low- to middle-income country
MNO: mobile network operator
