# Supplementary material for: Challenges and Potential Opportunities of Mobile Phone Call Detail Records in Health Research: Review
Source: JMIR Mhealth Uhealth. 2018 Jul 19;6(7):e161. doi: 10.2196/mhealth.9974 (PMC6072975; doi:10.2196/mhealth.9974)
Supplement: Multimedia Appendix 1 [file mhealth_v6i7e161_app1.pdf]

Appendix 1. Summary of included studies.

**Author/Year published**	**Country of focus**	**Using the data in conjunction**	**Findings**	**Information Governance**
Agarwal *et al*, 2013 [24]	Ivory Coast	CDR location data to derive human mobility	Mathematical model developed for disseminating emergency information during an epidemic	Orange Data4Development Ivory Coast Information Governance (IG)protocol (described in the narrative below)
Arai *et al*, 2013 [25]	Ivory Coast	**African socio-economic statistical survey datasets** (including HIV blood test results and demographic health surveys) were subsampled and a **GIS map of Ivory Coast** was overlaid on to these. Data were aggregated at the department level – 108 subdivisions of Ivory Coast. CDR data (number of calls and duration of calls) to determine connectivity between users was also aggregated to the department level and based on average number of calls. Comparisons were made between departments of high and low levels of HIV positive population, their demographics, income level and call volumes	Highlighted possible educational interventions for tackling HIV by using the derived network models to disseminate information	Orange Data4Development Ivory Coast IG protocol
Azman *et al*, 2013 [26]	Ivory Coast	**Human population density estimates** and size and duration of cholera epidemics (Afripop study).**Meteorological data**: flooding risk (Global Data Assimilation System) and rain estimates (Tropical Rainfall Measurement Mission).<br>High resolution CDRs (two week periods) were used to model people's travel frequencies and durations	Cholera transmission model developed based on human movement which can reproduce aspects of cholera epidemics including transmission hotspots	Orange Data4Development Ivory Coast IG protocol

Baldo & Closas, 2013 [27]	Ivory Coast	**Total number of influenza cases** detected from virological data (FluNet for Ivory Coast from the National Influenza Centres of the Global Influenza Surveillance and Response System).<br>CDRs (high spatial resolution) within hospital catchments.<br>Correlation between influenza numbers and CDR volumes were calculated	Identified key requirements for data needed to model disease outbreak systems due to the lack of granularity in the data provided to authors	Orange Data4Development Ivory Coast IG protocol
Bentgosson *et al*, 2011 [28]	Haiti	**Haiti population sizes by administrative division** for 2009. CDR data gave the estimated number of phone users per location for each administrative area, extrapolated to correspond to the population total, and compared with population sizes for the same periods	Estimates of population movements during disasters and outbreaks can be delivered rapidly and with potentially high validity in areas with high mobile phone use	Data anonymised by mobile network operator. Study approved by ethics board, Stockholm, Sweden
Bentgosson *et al*, 2015 [29]	Haiti	Researchers split Haiti into 78 study areas. If each area had 5 or more cases of cholera reported in a day, this was considered an outbreak **(data from National Cholera surveillance system)**. CDR location data were used to estimate the number of people moving from one study area to another.	CDR-based model developed by the study performed better than the optimized gravity models to predict the evolution of the 2010 Haiti cholera epidemic	Data anonymised by mobile network operator
Chunara & Nsoesie, 2013 [30]	Ivory Coast	**Anonymized data on the location of Ivory** Coast **disease alerts** for meningitis (Healthmap) were used to map when and to where the disease was spreading on a weekly basis – this was compared with mobility data derived from CDR location data and to verify the mathematical model	New way proposed by CDR data can be used to study disease dynamics and inform disease control measures	Orange Data4Development Ivory Coast IG protocol

Deka & Vishwan, 2013 [31]	Ivory Coast	CDR location data used to derive human mobility and social networks	A generative framework for understanding cellular social networks in relation to infection propagation	Orange Data4Development Ivory Coast IG protocol
De Monasterio *et al*, 2016 [32]	Argentina/ Mexico	CDR location data used to derive human mobility patterns	Method developed to tag human mobility on risk maps so health campaign managers can target specific areas and allocate resources more effectively	Argentinian dataset – anonymised by researchers<br><br>Mexican dataset – anonymised by mobile phone operator<br><br>Both datasets were aggregated by researchers
Dewulf *et al*, 2016 [33]	Belgium	Average pollution values were calculated for different areas of Belgium from **air quality measurements** (Belgian Interregional Environmental Agency), and CDR location data were used to measure how many people were present in these areas in order to calculate the levels of exposure	Daily exposure to air pollution can be calculated taking into individual travel patterns using CDRs	Anonymised by mobile phone operator. Individual exposure measures aggregated to postal code level
Djotio Ndie *et al*, 2013 [34]	Ivory Coast	Number of calls and texts from CDR data were compared with **HIV prevalence levels** in the subdivisions of Ivory Coast	Correlations were found between communication patterns and pathology prevalence	Orange Data4Development Ivory Coast IG protocol
Enns & Amuasi, 2013 [35]	Ivory Coast	Movements between sub-prefectures (**population density** at the level of sub-prefectures in Ivory Coast (1998 population census)) calculated by CDR location data were compared with malaria levels of the same areas over 5 months	Targeting vector control and behavioural change campaigns in sub-prefectures of high mobility and communication may be a more cost-effective	Orange Data4Development Ivory Coast IG protocol

			means of reducing national malaria prevalence	
Farrahi *et al*, 2014 [36]	Belgium	CDR mobility data and calls between users used to model social networks and the spread of infectious disease	Mobile phones found to be a viable option for health policies to arrest contagious diseases at the start of outbreaks	Institutional Review Board approval by the Massachusetts Institute of Technology Committee on the Use of Humans as Experimental Subjects, with written consent from the 72 participants
Finger *et al*, 2016 [37]	Senegal	Mobility patterns were extracted from CDR location data, and using the population data, the proportion of **populations counts** per region of Senegal (2005) (Afripop study) who were travelling during the observation time was calculated. This was then compared with **precipitation levels** (National Oceanic and Atmospheric Administration) and the **number of cholera cases** (WHO dataset/Senegalese Ministry of Health)	Mobile phone data showed that mass gatherings influence the course of a cholera epidemic	Data anonymised and aggregated by mobile network operator
Frias-Martinez *et al*, 2013 [38]	Mexico	CDR location data used to derive population movement patterns	Mobile phone data were able to simulate the spread of the H1N1 flu virus	No mention of any IG processes
Frias-Martinez *et al*, 2014 [39]	Mexico	**Dates of flu alerts** given by the Mexican government during the H1N1 flu outbreak April/May 2009 were used to identify whether these had an effect on individuals' behaviour (as analysed via CDR location data (population movement))	Mobile phone records demonstrated the impact of Mexican government restrictions during the H1N1 outbreak of April 2009	No mention of any IG processes

Gariazzo *et al*, 2016 [40]	Italy	Demographic data from Telecom Italia (gender, age range) were used to profile the mobile phone users. Census data was used to understand the **static population** and was compared with the mobility data derived from CDRs and **air pollution data**	Spatial and temporal accuracy of air pollution exposure assessment increased using mobile phone data	Anonymized by mobile network operator
Gavric *et al*, 2013 [41]	Ivory Coast	Mobility data and communication levels were derived from CDRs, and these were compared with **HIV prevalence rates** for each administrative area for Ivory Coast (from the Ministry of Fight against AIDS and the National Institute of Statistics and United States Census Bureau)	Key elements were identified that impact the rate of HIV infections and explain the spatial structure of epidemics	Orange Data4Development Ivory Coast IG protocol
Guzetta *et al*, 2014 [42]	Senegal	Mobility patterns were derived using the CDR data. Tuberculosis epidemiology was predicted using these patterns and compared with actual numbers from **tuberculosis prevalence, incidence and mortality** 2008-2012 (World Health Organization) to test the model	Model developed to evaluate the impact of human mobility patterns on the epidemiology of TB	Orange Data4Development Senegal IG protocol
Hamaoka, 2014 [43]	Senegal	CDR data provided hourly volume of incoming and outgoing calls and texts for each tower; anomalies were defined as deviations from regular call volume. These were compared with **event data** (information on holidays, natural disasters, political events (Japanese Embassy in Senegal)) to identify if there were similarities in dates of events with call anomalies	Model on how best a community should be organised and respond to anomalies such as epidemiological outbreaks and natural disasters	Orange Data4Development Senegal IG protocol
Kafsi *et al*, 2013 [44]	Ivory Coast	**Population data** (from the Afripop project) were compared with mobility levels derived from CDR	Model developed which simulates the behavioural	Orange Data4Development Ivory Coast IG protocol

		location data to understand the volume of travel for each sub-region of Ivory Coast	patterns of infected populations and enables personalised strategies to reduce infections	
Le Menach *et al*, 2011 [45]	Zanzibar	CDR location data was used to calculate the length of stay of visitors to Tanzania. This was compared with **ferry traffic data** (Zanzibar Ministry of Communication and Transport, 2006-2007) to understand the level of movement to and from the country. Results were compared with **Malaria surveys** (Zanzibar 2007)	Mobile phone data used to refine the estimate of malaria importation rates	Data anonymised by mobile network operator
Leidig *et al*, 2013 [46]	Ivory Coast	CDR location data to model human movement	Model developed to predict contagion diffusion which can be used to identify individuals for preventative measures	Orange Data4Development Ivory Coast IG protocol
Leidig *et al*, 2014 [47]	Senegal	**Employment data** for Senegal (working age population and percentage of employment) were used to verify calculations of work and home locations derived from CDR location data	Model developed to simulate Ebola virus diffusion which could inform Government prevention policies	Orange Data4Development Senegal IG protocol
Lima *et al*, 2013 [48]	Ivory Coast	CDR location data and communication patterns to account for user mobility and information spreading	Restricting mobility does not delay the occurrence of an epidemic	Orange Data4Development Ivory Coast IG protocol
Lima *et al*, 2014 [49]	Senegal	CDRs location data to derive human mobility	Using mobile phone data to model the transmission of infectious disease can quantify the risk	Orange Data4Development Senegal IG protocol

			of further spread and secondary infections	
Mari *et al*, 2014 [50]	Senegal	**Population data** (from the Afripop project) for each arrondissement was used to calculate resident human population. Human mobility data was calculated using CDR location data, and home location was also derived from these. This was compared with the georeferenced data on use of improved water sources and sanitation facilities (Global Atlas of Helminth Infections)	Modelling mobile phone data can be used to reproduce regional patterns of schistosomiasis	Orange Data4Development Senegal IG protocol
Matamalas *et al*, 2014 [51]	Senegal	Human mobility data (CDR location data) was compared with **geo-localized 'tweets'** to understand the level of Twitter activity for each arrondissement to gauge the effectiveness of using Twitter as a way to deliver interventions. **Demographic data** (from the Senegal data portal) were used to inform estimates on likelihood of contracting a disease	Using mathematical modelling mobile phone data can be used to track an epidemic outbreak	Orange Data4Development Senegal IG protocol
Milusheva, 2014 [52]	Senegal	Human mobility data (CDRs) was compared with malaria prevalence figures (Programme national de Lutte Contre le Paludisme) to gain an understanding on when and where the disease spread	Significant correlation between people returning from visits to places with high malaria prevalence and malaria prevalence in the home district	Orange Data4Development Senegal IG protocol
Mutafungwa, 2014 [53]	Senegal	Additional datasets were used to calculate the average distance from an individual's home to the closest hospital:<br>- **Administrative**	Mobile phone data combined with census data were used to estimate distance to	Orange Data4Development Senegal IG protocol

		**organization of Senegal**<br>- **Antenna GPS coordinates**<br>- **Senegal General Population and Housing Census National Agency of Statistics and Demographics**<br>- **List of hospitals – Online Senegal Medical Director and SenDoctor website.**<br>- **Data from OpenStreetMap.org**	hospital to inform public health strategies for people at risk	
Perez-Saez *et al*, 2014 [54]	Senegal	Home locations of mobile phone users were calculated via CDR location data. Road network data was used to calculate travel times between arrondissements	Using mathematical modelling, mobile phone data can be used to infer mobility patterns and the spread of waterborne infectious diseases	Orange Data4Development Senegal IG protocol
Resch *et al*, 2016 [55]	Austria	CDR location data used was used with **street data** (Open Street Map) to assess hospital catchment mapping	Mobile phone data can be used to delineate hospital catchment areas	Mobile phone data anonymised by mobile network operator
Saravanan *et al*, 2013 [56]	Ivory Coast	CDRs location data to derive human mobility patterns	A model is proposed to control the spread of communicable diseases by taking into account the spatial and temporal variations in human mobility patterns	Orange Data4Development Ivory Coast IG protocol
Tatem *et al*, 2014 [57]	Namibia	Human mobility was calculated using CDR location data and compared with **malaria incidence data** (Namibia national vector	Mobile phone data can identify areas where malaria surveillance	Ethical approval by University of Southampton. Anonymized records provided

		borne diseases control programme)	should be increased	by the mobile network operator were aggregated to the level of cell towers to preserve individual privacy.
Tatem et al, 2009 [58]	Zanzibar	Human mobility was calculated using CDR location data and **population distribution** data (Afripop project), and was compared with **malaria incidence data** (using a Global map of malaria endemicity – World malaria map (2007))	Mobile phone data can provide information on human mobility and malaria endemicity	Mobile phone data anonymised my mobile phone operator
Tompkins & McCreesh, 2014 [59]	Senegal	CDR location data	Characterisation of journeys that result in overnight stays which are considered relevant for malaria transmission	Orange Data4Development Senegal IG protocol
Tompkins & McCreesh, 2016 [60]	Senegal	CDR location data	In relation to malaria risk, the probability of a journey needing an overnight stay is highest at distance of 56km in Senegal	Orange Data4Development Senegal IG protocol
Vogel, 2014 [61]	Ivory Coast	Human mobility was calculated using CDRs and compared with malaria incidence data	Mobility patterns can be used to inform quarantine decisions	Orange Data4Development Ivory Coast IG protocol
Wesolowski & Buckee, 2013 [62]	Ivory Coast	CDR location data was used to derive models of population movement	Gravity models developed to understand how human mobility contributes to malaria transmission in Ivory Coast	Orange Data4Development Ivory Coast IG protocol
Wesolowski *et al*, 2015 [63]	Kenya	CDR location data was used to quantify daily travel patterns. **Birth rates** (2008–2009 Demographic Health Survey in Kenya) were	Captured seasonal human movement patterns relevant to	Mobile phone data anonymized by mobile network operator. Data were aggregated to

		compared with **Rubella incidence** (Rubella incidence system of general surveillance for measles). **Population data** (from WorldPop, 2009) was used to calculate the population of each administrative area	understanding the spread of childhood infectious diseases	either the province or district level scale.
Wesolowski *et al*, 2012 [64]	Kenya	Human mobility was calculated using CDR location data and compared with data from a 2009 **malaria prevalence** map	Identification of malaria importation routes	No details provided regarding IG processes
Wesolowski *et al*, 2014 [65]	Kenya	**Travel survey data** (malariometric community survey) was used with CDR mobility data during analysis	Combining datasets provides insights to model travel in low-income countries and understand the spread of infectious diseases	The community surveys were conducted and approved by the ethical committees of the London School of Hygiene and Tropical Medicine (LSHTM) and the Kenya Medical Research Institute (KEMRI).<br><br>Mobile phone data anonymized by mobile network operator and their use approved by Harvard University Institutional Review Board.
Wesolowski *et al*, 2015 [66]	Pakistan	CDR location data and population data (WorldPop, 2009) were used to calculate human mobility patterns. These were compared with the **incidence rates for Dengue disease data** (cases reported by hospitals, health clinics, and labs)	Human mobility derived from mobile phone data predicts epidemic patterns in Pakistan and the spatial extent and timing of dengue disease outbreaks	Dengue data de-identified and aggregated to the tehsil level. Fuller data governance details are given in the narrative below.
Wilson *et al*, 2016 [67]	Nepal	CDRs location data used to derive human mobility	Mobile phone records can provide information on the mobility of	Analysis was undertaken in compliance with the GSMA privacy guidelines

			people after a natural disaster in a short time frame which can inform humanitarian response	developed in the context of the Ebola outbreak. Data governance is described in the narrative below.
Wu *et al*, 2014 [68]	Senegal	CDR location data used to derive human mobility	Model developed for seeding parameters for an epidemiological model to study infectious diseases	Orange Data4Development Senegal IG protocol
Yi Yu, 2014 [69]	Senegal	CDR location data to derive mobility data of users in relation to other users	Model developed to predict the likelihood of infectious disease transmission and its progression	Orange Data4Development Senegal IG protocol
